# Graph Constraint and Collaborative Representation Classifier Steered Discriminative Projection with Applications for the Early Identification of Cucumber Diseases

**DOI:** 10.3390/s20041217

**Published:** 2020-02-23

**Authors:** Yuhua Li, Fengjie Wang, Ye Sun, Yingxu Wang

**Affiliations:** College of Engineering, Nanjing Agricultural University, Nanjing 210031, China; 2019212011@njau.edu.cn (F.W.); sunye@njau.edu.cn (Y.S.); 2017112018@njau.edu.cn (Y.W.)

**Keywords:** cucumber disease identification, hyperspectral imaging, discriminative projection, collaborative representation, graph constraint

## Abstract

Accurate, rapid and non-destructive disease identification in the early stage of infection is essential to ensure the safe and efficient production of greenhouse cucumbers. Nevertheless, the effectiveness of most existing methods relies on the disease already exhibiting obvious symptoms in the middle to late stages of infection. Therefore, this paper presents an early identification method for cucumber diseases based on the techniques of hyperspectral imaging and machine learning, which consists of two procedures. First, reconstruction fidelity terms and graph constraints are constructed based on the decision criterion of the collaborative representation classifier and the desired spatial distribution of spectral curves (391 to 1044 nm) respectively. The former constrains the same-class and different-class reconstruction residuals while the latter constrains the weighted distances between spectral curves. They are further fused to steer the design of an offline algorithm. The algorithm aims to train a linear discriminative projection to transform the original spectral curves into a low dimensional space, where the projected spectral curves of different diseases own better separation trends. Then, the collaborative representation classifier is utilized to achieve online early diagnosis. Five experiments were performed on the hyperspectral data collected in the early infection stage of cucumber anthracnose and *Corynespora cassiicola* diseases. Experimental results demonstrated that the proposed method was feasible and effective, providing a maximal identification accuracy of 98.2% and an average online identification time of 0.65 ms. The proposed method has a promising future in practical production due to its high diagnostic accuracy and short diagnosis time.

## 1. Introduction

Low temperature, scant lighting, high humidity and other extremely complicated greenhouse environments frequently cause cucumber diseases. Moreover, most diseases spread rapidly. Thus, accurate and rapid identification of diseases in the early stage of infection has great practical significance. Traditional methods rely on naked eye observation [1,2], pathologic analysis including the microscopic observation of pathogen morphology, as well as molecular, serological, and microbiological diagnostic techniques [3]. Because of the poor real-time performance and high requirement for professional analysts, pathologic analysis is rarely used in practical production [4]. As for naked eye observation, it lacks unified measurement criteria to go on and is influenced by the observer’s subjective consciousness and empirical knowledge, which often results in a wrong diagnosis. Moreover, due to the resolution ratio of the human eye, it is almost impossible to distinguish diseases only by naked eye especially at the early stage of infection.

With the rapid development of computer vision and artificial intelligence, visual-image (composed of three wavelength bands: 475, 520 and 650 nm) processing technique has been successfully exploited for disease diagnosis [5,6,7,8]. The earliest study can date back to the mid-1980s. In 1985, Yasuoka et al. [9] researched the infrared image of crop blades polluted by noxious gas. Since then, plant disease diagnosis by analyzing the image of diseased blade started. Based on the optical filtering and spectroscopic characteristics on healthy and diseased leaves, Sasaki et al. [10] established identification parameters using a genetic algorithm and studied the automatic diagnosis of cucumber anthracnose. El-Helly et al. [11] developed an image processing system to automatically detect disease spots and well-differentiated cucumber downy mildew and powdery mildew diseases using an artificial neural network. Geng et al. [12] analyzed the mean distribution of Cb and Cr channels in YCbCr space, and effectively separated the information pertaining to cucumber downy mildew by constructing an algorithm combining Cb and Cr channels. Peng et al. [13] extracted the color and texture features of cucumber blades and established a linear discriminant model for cucumber downy mildew and anthracnose. To effectively reduce the computation cost and improve the identification performance, Zhang et al. [14] segmented diseased blades by *K*-means clustering, extracted shape and color features from lesions, and realized the diagnosis using sparse representation classifiers. Their success suggests that the visual-image processing technique has great potential in plant disease diagnosis. However, their effectiveness depends on obvious symptoms. In other words, they work well only with obvious disease spots containing the information of color, shape, texture, etc. But at the early infection stage, disease symptoms are often unobvious, and visual-image-based methods struggle to work in such a situation. 

Unlike the common methods above, hyperspectral imaging (HSI) technique obtains both the spatial and spectral information of plants over a large range of the light spectrum, which has shown significant potentials and advantages for identifying plant diseases [2,3,15]. As we know, after infection, changes in plant tissues occur earlier than disease symptoms and can be reflected by the radiation to electromagnetic waves. Given that, HSI technique can be utilized to detect diseases based on the variations of reflectance spectra, even if symptoms are unobvious. Over the past few years, many HSI-based methods and systems have been developed, which can be roughly divided into two categories: feature-extraction-based method and the effective-wavebands-based method. The latter category also includes methods on reflectance indices obtained by combining the effective wavebands. One of the best reflectance indices is the photochemical reflectance index (PRI), introduced by Gamon et al. [16], which can show stress-induced changes in photosynthesis [17]. Though reflectance indices can simplify the analysis of the reflectance spectrum, they can be affected by many factors, such as illumination, atmosphere, soil background and location [17,18]. Compared with the reflectance indices with fixed calculation formulas, feature-extraction-based methods have the advantage that one can autonomously design appropriate algorithms to extract features that are invariant to interferences such as illumination variations and atmospheric noise to a certain degree. Below, we introduce some effective feature-extraction-based methods. Ma et al. [19] proposed an identification method for Fusarium head blight by applying continuous wavelet analysis to the reflectance spectra of wheat ears. Chai et al. [20] proposed rapid identification of cucumber diseases based on HSI and distance discriminant analysis. Barbedo et al. [21] presented an automatic method to detect the fusarium head blight disease in wheat kernels by performing morphological mathematical operations and spectral band manipulations on hyperspectral data. Based on the HSI technique, Cen et al. [22] detected chilling injury in cucumbers by combining three feature-extraction methods with two traditional classification methods, and achieved the overall accuracy of 90.5%. Zhu et al. [23] utilized machine learning classifiers and variable selection methods to research the potential of pre-symptomatic identification of tobacco disease. Although the HSI technique has the capability to detect diseases at a much earlier infection stage, the vast majority of current studies are still concentrated on the cases with obvious lesions. 

Consequently, this paper aims to establish an early identification method for cucumber diseases based upon HSI technique. By analyzing the reflectance spectra of diseased and normal leaves, it can be observed that the spectral curves of different diseases have a certain degree of similarity in appearance and shape; besides, the coverage areas of the spectral curves corresponding to different diseases are almost coincident. Therefore, it is very difficult to distinguish diseases in the original hyperspectral data space. Moreover, hyperspectral data are generally of high dimensionality and direct processing may result in high computation and time costs. To address such problems, this study attempts to train a discriminative projection to transform the spectral curves into a low dimensional space, in which the similarity of spectral curves of the same disease is enhanced while that of the different diseases is weakened. However, even if the above goal is achieved, the projection does not necessarily guarantee a positive impact on the ultimate goal because the training procedure is completely independent of the subsequent diagnosis. To address this problem, we establish a connection between them by utilizing the decision rule of the collaborative representation classifier (CRC) [24] to steer the training procedure. Since the label and spatial distribution information of the data is usually of great importance for discrimination [25], we additionally design graph constraints to steer the training procedure. In summary, this paper presents a graph constraint and CRC-steered discriminative projection learning method (CRC-DP) and applies it to the early identification of cucumber diseases.

## 2. Materials and Methods

### 2.1. Acquiring the Hyperspectral Data

‘Lufeng’ cucumber is a widely cultivated cucumber variety because of its strong growth vigor and resistance to diseases such as downy mildew, powdery mildew and fusarium wilt. Herein, it was used for experiments. A total of 55 healthy cucumber plants were selected. Their age was 36 days. All the selected plants were of a similar growth condition and had three leaves. Among these, 25 plants were randomly selected for inoculation against cucumber anthracnose; another 25 plants were inoculated against cucumber *Corynespora cassiicola*; and the above 50 plants formed the inoculation group; the remaining 5 healthy plants formed the healthy control group. The strains were purchased from the agricultural culture collection of China. Inoculation was conducted by manually making a small cut on the leaf using a sharp knife and then covering the cut with a small mycelia block. Two leaves were inoculated for each plant. After inoculation, plants of different groups were put in different artificial climate boxes for cultivation. The artificial environment was controlled with a relative humidity of 90% and temperatures of 28 °C and 24 °C respectively for day and night. The illumination and darkness durations were set to 16 h and 8 h, respectively. LED lights with illuminance of 22,000 lx were used to provide illumination at cultivation. About 24 h later, hyperspectral images of 100 inoculated leaves in the inoculation group and two normal leaves of each plant in healthy control group were acquired every 24 h using a push-broom HSI system named GaiaSorter (Dualix spectral imaging, Chengdu, China). HSI images stopped being collected after 12 days. Hence, there were 1320 hyperspectral images with each image containing one leaf. The HSI system comprised two hyperspectral imaging units (visible and near infrared), a horizontal motorized translation stage (HSIA-T1000), image acquisition software (SpecView), and a uniform illumination light source (HSIA-LS-T-H), which was composed of 8 halogen lamps with adjustable light intensity and provided spectra of 350–2500 nm. In this paper, we only used the visible hyperspectral imaging unit to collect raw hyperspectral images, which consisted of 256 spatially resolved reflectance profiles with 1394 × 1024 pixels for the wavelengths of 391 to 1044 nm with a spectral resolution of 2.8 nm. Leaves with lesions occupying less than 20% of the leaf area were selected for experiments. 

Affected by the measurement environment, the status of experimental devices, the skill level of operators and other factors, the collected hyperspectral images often contained some noise and disturbing information. To alleviate their adverse effects, a correction was performed using the following formula:

(1) $$I=\frac{I_{O}-I_{D}}{I_{W}-I_{D}}$$

where, $I_{O}$ and I respectively represents the hyperspectral image before and after correction; $I_{D}$ is a dark reflection image obtained when the halogen lights are turned off and the camera lens is completely covered with its own non-reflective opaque black cap with 0% reflectance; $I_{W}$ is a white reflection image obtained by capturing the hyperspectral image of a Teflon white board with 99% reflectance. Afterwards, the spectral curves of pixels within disease lesions were extracted for further analysis.

### 2.2. Proposed CRC-DP Method

As stated in the introduction, identifying different diseases directly in the original hyperspectral data space is difficult. Thus, we aimed to locate a low-dimensional space in which the projected spectral curves of different diseases can be well separated. Here, for narrative convenience, we take each vectorized spectral curve as a sample and refer to ‘cucumber anthracnose disease’, ‘cucumber *Corynespora cassiicola* disease’ and ‘normal plant’ as the first, second and third class of disease, respectively. The CRC-DP method consists of two sequential procedures, which are respectively described in detail as follows.

#### 2.2.1. Offline Training Stage

Suppose each class has enough training samples spanning a subspace and any sample from this class lie on this subspace. Let $X=\left[x_{1},\cdots ,x_{k}\right]\in R^{n\times k}$ represent all the training samples in the high-dimensional input space, where $x_{i}$ is the $i^{th}$ training sample and k is the number of training samples. ${\left\{x_{i}\right\}}_{i=1}^{k}$ are linearly converted to new ones in low-dimensional space by $y_{i}=P^{T}x_{i}\left(i=1,\cdots ,k\right)$, where $P\in R^{n\times m}$ is the desired discriminative projection matrix. According to a modified collaborative representation model, each training sample in the low-dimensional space is encoded as a linear combination of the rest training samples by Equation (2):

(2) $$\begin{array}{c} \underset{w_{i}^{\prime }}{\arg\min}{\left\|y_{i}-\left(w_{i,1}^{\prime }y_{1}+w_{i,2}^{\prime }y_{2}+\cdots +w_{i,k}^{\prime }y_{k}\right)\right\|}_{2}^{2}=\underset{w_{i}^{\prime }}{\arg\min}{\left\|y_{i}-Yw_{i}^{\prime }\right\|}_{2}^{2}\\ s.t.\left\{ \begin{array}{l} 1^{T}w_{i}^{\prime }=1\\ w_{i,i}^{\prime }=0 \end{array}\right. \end{array}$$

where $Y=P^{T}X=[y_{1},\cdots ,y_{k}]$; the collaborative representation coefficient vector $w_{i}^{\prime }={\left[w_{i,1}^{\prime },\cdots ,w_{i,k}^{\prime }\right]}^{T}$ is a *k*-dimensional column vector whose $i^{th}$ element is forced to zero; $1\in R^{k\times 1}$ is a column vector consisting of all ones. Obviously, Equation (2) can be considered as a least-square problem and thus has an analytical solution. Since the negative coefficients in $w_{i}^{\prime }$ have no practical significance, they are further updated using Equation (3):

(3) $$w_{i,j}=\left\{ \begin{array}{ll} 0, & \mathrm{if}\;w_{i,j}^{\prime }\leq 0\\ w_{i,j}^{\prime }, & \mathrm{if}\;w_{i,j}^{\prime }>0 \end{array}\right.$$

By doing this, a new coefficient vector $w_{i}={\left[w_{i,1},\cdots ,w_{i,k}\right]}^{T}$ can be obtained. 

Based on the decision rule of CRC, each sample from the $c^{th}$ class should be well represented by the training samples from the $c^{th}$ class. To this end, a same-class reconstruction residual is defined as:

(4) $$\frac{1}{k}\sum_{i=1}^{k}{\left\|y_{i}-Y\delta_{c}\left(w_{i}\right)\right\|}_{2}^{2}=tr\left(P^{T}R_{s}P\right)$$

where $R_{s}=\left(1/k\right)\sum_{i=1}^{k}\left(x_{i}-X\delta_{c}\left(w_{i}\right)\right){\left(x_{i}-X\delta_{c}\left(w_{i}\right)\right)}^{T}$ and $\delta_{c}\left(w_{i}\right)$ is a column vector obtained by preserving the entries of $w_{i}$ associated with the $c^{th}$ class and setting the rest to zeros. Beyond that, training samples from the $s^{th}\left(s\neq c\;\mathrm{and}\;s\in \left\{1,2,\cdots ,M\right\}\right)$ class should not be able to well represent this sample. To this end, we define a different-class reconstruction residual as:

(5) $$\frac{1}{k\left(M-1\right)}\sum_{i=1}^{k}\sum_{s\neq c,s=1}^{M}{\left\|y_{i}-Y\delta_{s}\left(w_{i}\right)\right\|}_{2}^{2}=tr\left(P^{T}R_{d}P\right)$$

where $R_{d}=\left(1/k\left(M-1\right)\right)\sum_{i=1}^{k}\sum_{s\neq c,s=1}^{M}\left(x_{i}-X\delta_{s}\left(w_{i}\right)\right){\left(x_{i}-X\delta_{s}\left(w_{i}\right)\right)}^{T}$ and M is the number of classes. The above two reconstruction residuals are named as discriminative reconstruction fidelity terms. To meet the decision rule of CRC, the same-class reconstruction residual is imposed to be as small as possible while the different-class reconstruction residual is imposed to be as large as possible. The discriminative fidelity terms are powerful for both representation and classification but fail to take into consideration the spatial distribution and label information of the training samples, which are of great importance for classification.

To solve the above problem, we introduce two novel graph constraints to associate the class labels with the spatial distributions of training samples. First of all, a same-class graph Η and a different-class graph G are respectively constructed as follows:

(6) $$H_{i,j}=\left\{ \begin{array}{ll} 1, & if\;l\left(i\right)=l\left(j\right)\\ 0, & otherwise \end{array}\right.$$

(7) $$G_{i,j}=\left\{ \begin{array}{ll} 1, & if\;l\left(i\right)\neq l\left(j\right)\\ 0, & otherwise \end{array}\right.$$

where l(i) denotes the class label of $x_{i}$ with i=1,2,⋯,k. Η reflects the relation of samples belonging to the same class while G reflects the relation of samples belonging to different classes. To ensure samples from different classes can be well separated, the CRC-DP method encourages that in the low-dimensional space, if two training samples are from the same class, they should reside close to each other, and if two training samples are from different classes, they should be far away from each other. To this end, a same-class graph constraint and a different-class graph constraint are, respectively, mathematically formulated as:

(8) $$\sum_{i=1}^{k}\sum_{j=1}^{k}{\left\|y_{i}-y_{j}\right\|}_{2}^{2}H_{i,j}=tr\left(P^{T}XLX^{T}P\right)$$

(9) $$\sum_{i=1}^{k}\sum_{j=1}^{k}{\left\|y_{i}-y_{j}\right\|}_{2}^{2}G_{i,j}=tr\left(P^{T}XZX^{T}P\right)$$

where L=D−H and D is a diagonal matrix with entry $D_{i,i}$ the summation of the $i^{th}$ row of Η. Z=Q−G and Q is a diagonal matrix with $Q_{i,i}$ entry the summation of the $i^{th}$ row of G. Differing from the local-graph constraint proposed by Zheng et al. [26] which preserves the local (neighborhood) structure of data, the graph constraints force the training samples from the same class more concentrated and avoids parameter selection. To enhance the discrimination, we need to minimize the same-class graph constraint and maximize the different-class graph constraint.

Finally, we incorporate the fidelity terms with the graph constraints and formulate the objective function as:

(10) $$J\left(P\right)=\frac{tr\left(P^{T}R_{d}P\right)+tr\left(P^{T}XZX^{T}P\right)}{tr\left(P^{T}R_{s}P\right)+tr\left(P^{T}XLX^{T}P\right)}=\frac{tr\left(P^{T}UP\right)}{tr\left(P^{T}TP\right)}$$

where $U=R_{d}+XZX^{T}$ and $T=R_{s}+XLX^{T}$. The optimal projection matrix P can be determined by maximizing the objective function (Equation (10)). We impose a constraint $P^{T}TP=I$ on the objective function. By doing this, P can be formed by the generalized eigenvectors of Uφ=γTφ corresponding to the largest m eigenvalues. However, ${\left\{w_{i}\right\}}_{i=1,\cdots ,k}$ in $R_{d}$ and $R_{s}$ are unknown beforehand, so we solve P and ${\left\{w_{i}\right\}}_{i=1,\cdots ,k}$ in an iterative manner. P is initialized using a n×m random matrix and each iteration mainly includes four steps: (a) project ${\left\{x_{i}\right\}}_{i=1}^{k}$ to ${\left\{y_{i}\right\}}_{i=1}^{k}$ using $y_{i}=P^{T}x_{i}\left(i=1,\cdots ,k\right)$; (b) solve the collaborative representation coefficients ${\left\{w_{i}\right\}}_{i=1,\cdots ,k}$ using Equations (2) and (3); (c) compute $R_{d}$ and $R_{s}$; (d) obtain a new projection matrix P by maximizing the objective function (10). Repeat the above steps until the difference of the objective function values between two iterations is smaller than a predefined value ε. 

#### 2.2.2. Online Identification Stage

Given a query sample x whose identity (for disease identification, “identity” refers to the type of disease that the query sample is infected with) is unknown beforehand, we determine it as follows. Firstly, x is converted to a m×1 vector by $y=P^{T}x$. Then, we collaboratively represent y as $y=\alpha_{1}y_{1}+\alpha_{2}y_{2}+\cdots +\alpha_{k}y_{k}$ using all the training samples in the low-dimensional space. And the coefficient vector $\alpha ={\left[\alpha_{1},\alpha_{2},\cdots ,\alpha_{k}\right]}^{T}$ is obtained by solving a regularized least square problem:

(11) $$\underset{\alpha }{\arg\min}{\left\|y-\left(\alpha_{1}y_{1}+\alpha_{2}y_{2}+\cdots +\alpha_{k}y_{k}\right)\right\|}_{2}^{2}=\underset{\alpha }{\arg\min}{\left\|y-Y\alpha \right\|}_{2}^{2}\;\;\;s.t.\;\;1^{T}\alpha =1$$

The identity $j^{*}$ of x can be determined by evaluating which class of training samples leads to the minimal reconstruction residual, as follows:

(12) $$j^{*}=\underset{j}{\arg\min}{\left\|y-Y\delta_{j}\left(\alpha \right)\right\|}_{2}^{2}$$

**Figure 1:**
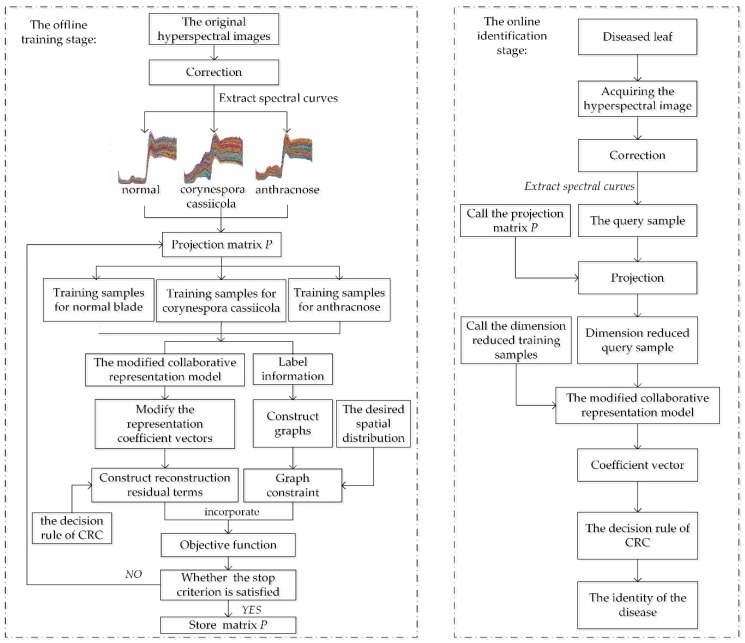
The flowchart of the CRC-steered discriminative projection learning method (CRC-DP).

To show our method more concisely, the overall framework and detailed steps of CRC-DP method is summarized as Algorithm 1 and a flowchart of CRC-DP method is plotted in Figure 1.
**Algorithm 1.** CRC-DP method.**Input:** the query sample x, the training samples $X=\left[x_{1},x_{2},\cdots ,x_{k}\right]\in R^{n\times k}$, parameters ε and m.**Offline training stage:**1. Initialize P using a n×m random matrix.If the values of objective function between two iterations is larger than ε, repeat steps 2–5.2. Project X to the m-dimensional space by $y_{i}=P^{T}x_{i}\left(i=1,2,\cdots ,k\right)$.3. Solve ${\left\{w_{i}\right\}}_{i=1,\cdots ,k}$ using Equations (2) and (3).4. Calculate $R_{d}$ and $R_{s}$.5. Update P using the generalized eigenvectors of Uφ=γTφ corresponding to the largest m eigenvalues.**Online identification stage:**1. Transform x by $y=P^{T}x$.2. Represent y as $y=\alpha_{1}y_{1}+\alpha_{2}y_{2}+\cdots +\alpha_{k}y_{k}$ and solve the coefficient vector α.3. Determine the identity of x by Formula (12).

### 2.3. Experiment Design and Setup

The proposed method consists of two parts: training a projection matrix to transform samples into a low-dimensional space and then identifying disease using the modified CRC. The former, if used as a dimension reduction (DR) operation, can be applied to classification problem (disease identification is also a classification problem). As we know, DR should be beneficial for the subsequent classification. In other words, the samples of different classes should have better separation trends after DR. Thus, to verify whether DR using the CRC-DP method can lead to better separation trends than using other DR methods, we first performed different DR methods on two types of easily accessible unitless data (a manually created toy dataset and wine dataset from UCI [27]) to project them to a low-dimensional space. Then, to evaluate our method’s capability in the early diagnosis of plant disease, some experiments are performed using the hyperspectral data collected in the early infection stage of cucumber anthracnose and cucumber *Corynespora cassiicola*. Herein, the training and testing sets are prepared as follows unless otherwise stated: 1000 hyperspectral curves were extracted from the lesions of each disease, among which, half were randomly selected for training and the rest were used for testing. As for normal leaves, 500 hyperspectral curves were extracted for training and testing, respectively. Each hyperspectral curve is vectorized by stacking the reflectance values of band 391–1045 nm. By doing this, each sample is a 256 × 1 column vector. After that, normalize it to have the unit *l*_2_-norm and then take it as a sample. Thus, the training and testing sets respectively have three classes of 1500 samples. Note that ‘normal’ was considered as the third type of disease for narrative convenience. For comparison, we also assessed the performances of five other classifiers: support vector machines (SVM), *K*-nearest neighbor classifier (KNN), naive Bayes classifier (NB), random forest classifier (RF) and discriminant analysis classifier (DA). 

Here, we briefly introduce the principles of these five classifiers:

- SVM seeks hyperplanes to classify samples in high-dimensional space. The goal of SVM is to maximize the margin between hyperplanes and support vectors, which can be solved by transforming into a convex quadratic programming problem.
- The core idea of KNN classifier is that if the majority of the K most-similar samples of a query sample belong to a certain category, then the query sample also belongs to this category. KNN does not require training.
- The principle of NB is to calculate the posterior probability of the query sample using its prior probability, and the query sample belongs to the class with the largest posterior probability.
- RF repeatedly randomly selects samples with placement from the original training set to generate a new training set to train decision tree, then repeat the above steps to train multiple decision trees to form a random forest. Given a query sample, each decision tree is used to make a decision and finally determine which category it belongs to by voting.
- Distance-based DA calculates the distance between the query sample and the mean of all the training samples of each class. Then, the query sample is classified into the class with the minimal distance.

According to experimental experiences, unless otherwise specified, parameter ε in CRC-DP method is set as 0.05; the number of neighbors in KNN and the number of decision trees in RF take values between 1 and τ−1 with intervals of 1 and 25 respectively, where τ represents the number of training samples per class. We report their best results here. All the experiments are carried out on a 2.1 GHz computer with 64 GB RAM.

## 3. Results and Discussion

### 3.1. Effects of Different DR Methods

**Figure 2:**
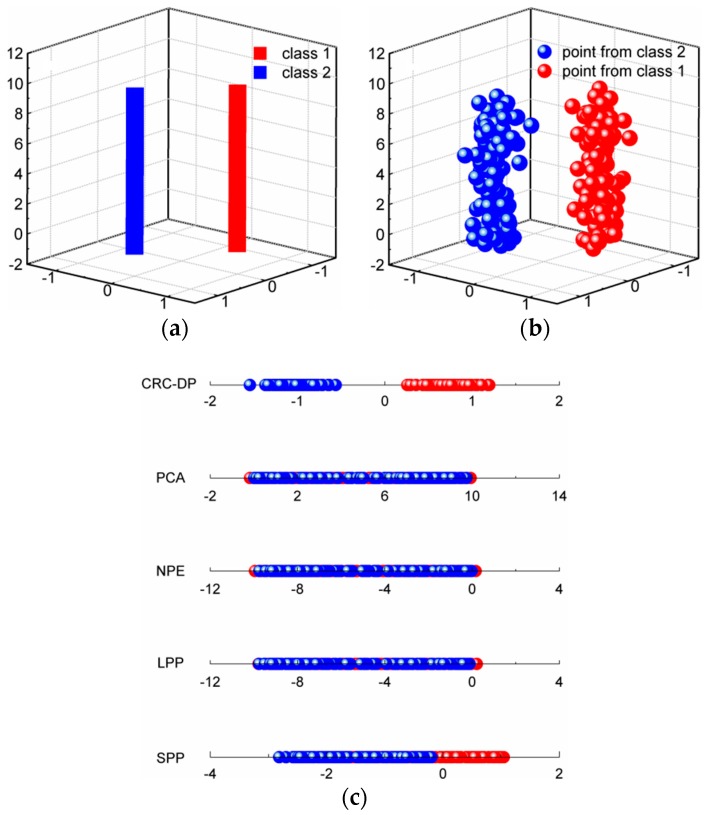
(**a**) Two classes of data; (**b**) 100 points from each class; (**c**) the one-dimensional results using different DR methods.

**Figure 3:**
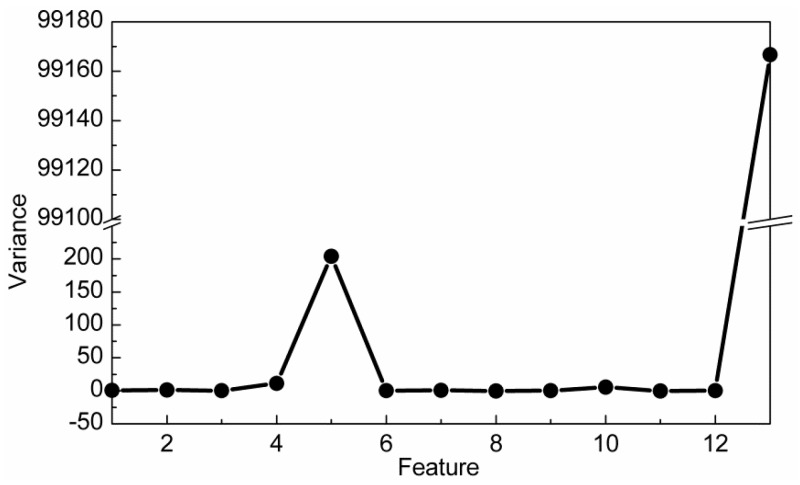
The variances of 13 features on the wine data.

**Figure 4:**
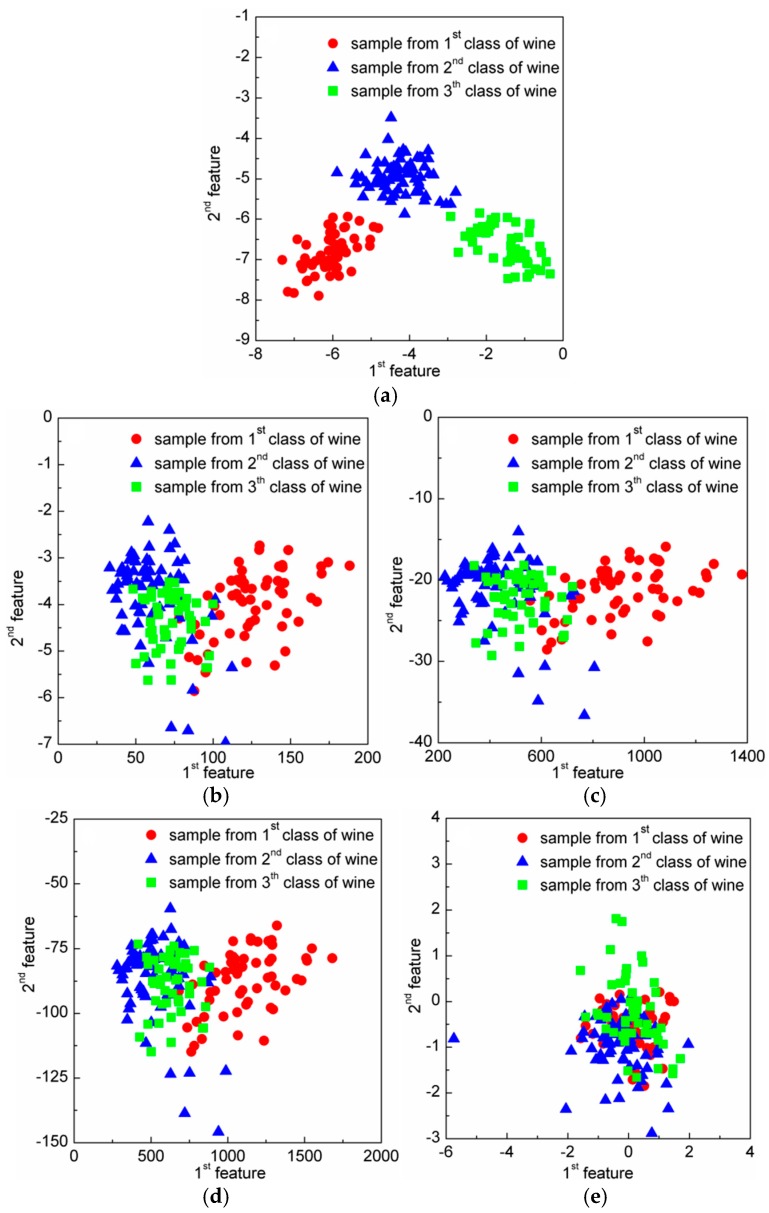
The two-dimensional results of the wine data using different dimension-reduction (DR) methods: (**a**) CRC-steered discriminative projection learning method (CRC-DP); (**b**) locality preserving projection (LPP); (**c**) neighborhood preserving embedding (NPE); (**d**) principal component analysis (PCA); (**e**) sparsity preserving projection (SPP).

In classification problems, DR should be conducive to the subsequent classification. In other words, samples among different classes should present good classification boundaries after DR, thus they can be easily and well separated by several hyperplanes. In order to show that DR using the CRC-DP method can lead to better separation trends than using other DR methods, two experiments are conducted in this section. (1) Toy dataset: similarly to Qiao et al. [25], we produced two classes of data points. As shown in Figure 2a, each bar denotes one class; 100 samples (points) were randomly selected from each class and are contaminated with Gaussian white noise with standard deviation of 0.15 to make them to appear in a 3-dimensional space (Figure 2b). Each sample has three features (the values corresponding to three axes) with variances 0.3051, 0.2633 and 8.3375 respectively. Apparently, the sample distribution mainly depends on the third feature due to its largest variance. Sparsity preserving projection (SPP) [25], principal component analysis (PCA) [28], neighborhood preserving embedding (NPE) [29], locality preserving projection (LPP) [30] and CRC-DP methods were respectively utilized to project the points shown in Figure 1b to a 1-dimensional space. The results are shown in Figure 2c. (2) Wine dataset from UCI: There are three classes of wine with 178 samples. And each sample has 13 features. The variances of 13 features are plotted in Figure 3, and it can be observed that the 13th feature has the largest variance. We apply SPP, PCA, NPE, LPP, and CRC-DP methods respectively to project the wine data to a 2-dimensional space. The results are shown in Figure 4. From Figure 2c and Figure 4, it can be seen that CRC-DP method outperforms other three-dimensional reduction methods and it not only separates samples of different classes well, but also constrains samples of the same class in a more concentrated manner in the low-dimensional space. The reason may be that CRC-DP method adopts graph constraints to steer the training of the projection, ensuring the projected samples of the same class are as close as possible while that of the different classes are as far away as possible. As a result, the projected samples in low-dimensional space present obvious classification boundaries. For NPE and LPP, the projected samples from different classes are mixed together because the Euclidean distance and the neighbor size fail to identify the real local structure they supposed [25]. As for PCA, it also fails to separate the samples. The reason is that PCA is aimed to maximize the variance of samples in the low-dimensional space. As analyzed earlier, the third feature of the toy data and the 13th feature of the wine data have the largest variances, thus they affect PCA the most. For SPP, there is also a number of projected samples aliasing. Both the above two experiments validate that CRC-DP method can lead to better separation trends than all the compared DR methods, which is beneficial for separation. In addition, the results suggest that one should seriously take the data distribution into consideration when selecting a DR method, otherwise an inappropriate DR method is not necessarily positive for good separation trends among different classes.

### 3.2. Early Identification of Cucumber Leaf Diseases

**Figure 5:**
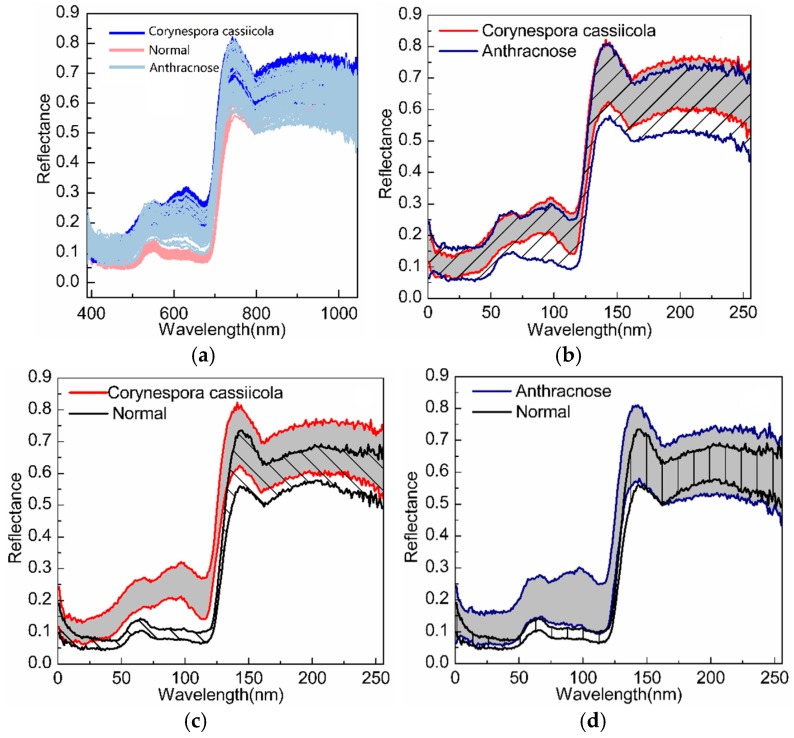
The coverage areas of spectral curves corresponding to different diseases: **(a**) normal, anthracnose and *Corynespora cassiicola*; (**b**) anthracnose and *Corynespora cassiicola*; (**c**) normal and *Corynespora cassiicola*; (**d**) normal and anthracnose.

In this section, we first analyze the characteristics of spectral curves of different diseases to show that it is difficult to directly distinguish different diseases in the original hyperspectral space. Then, experiments are conducted to classify three types of cucumber diseases (anthracnose, *Corynespora cassiicola* and normal) to verify the feasibility and capability of the CRC-DP method in disease early diagnosis. Figure 5 compares the coverage areas of spectral curves corresponding to different diseases, in which, the spectrum of cucumber anthracnose lesions, *Corynespora cassiicola*, lesions and normal blades are respectively within the area between the two blue lines, red lines and black lines. It can be seen that the spectrum of cucumber *Corynespora cassiicola* lesions is mostly covered by that of the cucumber anthracnose lesions; and the appearances of spectral curves of different diseases are very similar; the spectrum of wavelength bands 50–125 nm can be used to distinguish cucumber leaves’ infection by *Corynespora cassiicola* or anthracnose from normal ones since there is no overlap at all. The above phenomenon implies that it is difficult to directly distinguish cucumber anthracnose and *Corynespora cassiicola* in the original hyperspectral data space. To alleviate this problem as well as to reduce the computation and time costs, DR is conducted before classification. The CRC-DP method directly uses its discriminative projection matrix while other classifiers use PCA to reduce the sample dimension to *m*. All the following experiments implement three-class classification (‘anthracnose’, ‘*Corynespora cassiicola*’ and ‘normal’ correspond to the ‘first’, ‘second’ and ‘third’ class respectively).

**Figure 6:**
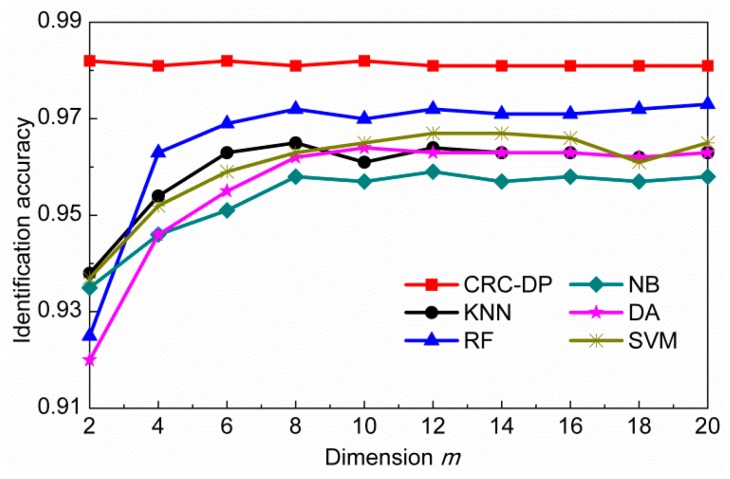
The identification accuracy versus the reduced sample dimension *m*.

**Figure 7:**
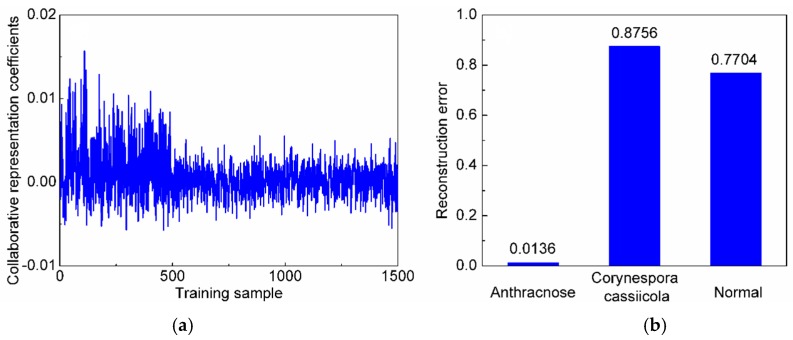
(**a**) The collaborative representation coefficients of a query sample from the first class; (**b**) the reconstruction residuals corresponding to each disease.

We initially conducted an experiment to measure the performances of different methods for three-class classification. Here, m is fixed as 10. Table 1 shows the classification accuracies and the number of incorrectly diagnosed samples of different methods. From Table 1, it can be seen that the CRC-DP method achieves higher classification accuracies than all the compared methods. After that, an experiment is carried out to assess the influence of the reduced sample dimension m and the results are plotted in Figure 6. From which, we can see that the CRC-DP method provides the highest classification accuracies in all cases and even in the case of extremely low dimension values such as m=2, it can still obtain high classification accuracy of 98.2%. The results validate that the CRC-DP method is robust to the reduced features dimension m to some extent. In practical applications, real-time diagnosis is especially important. Thus, an experiment was conducted to assess the mean online identification time of each query sample and the results are listed in Table 2. Here, the number of neighbors in the KNN classifier is set as 9 and the number of decision trees for random forest classifier is set as 200. Table 2 shows that except for random forest classifier, all other methods have online identification time less than one millisecond, which can meet the requirements of real-time applications. The results shown in Table 1 and Table 2 and Figure 6 validate that the CRC-DP method runs fast and achieves high identification accuracy even without conducting preprocessing and effective wavebands selection before diagnosis. Figure 7a shows the collaborative representation coefficients of a query sample from the first class (anthracnose), and the horizontal axis corresponds to the 1500 training samples from three classes. Figure 7b shows the reconstruction residuals corresponding to the three classes. It can be noted that the first class has the minimal reconstruction residual, thus the query sample is judged to the first class, which is consistent with the ground-truth. 

**Figure 8:**
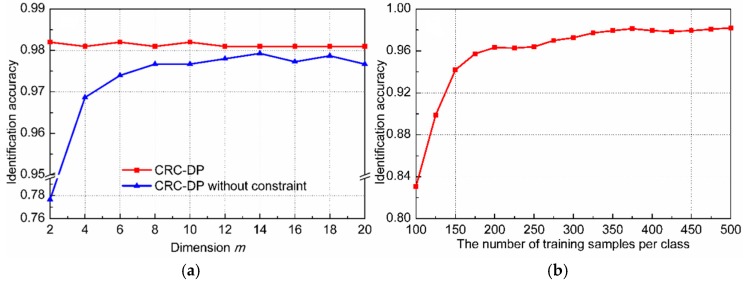
(**a**) Comparison results of the CRC-DP method with and without graph constraint; (**b**) identification accuracy versus the enrollment size by CRC-DP method.

There are some other factors that may affect the performance of CRC-DP method to varying degrees, such as the graph constraints and the number of training samples per class. Therefore, let us further examine their influences using the following experiments, in which, the reduced dimension m of each sample is fixed as 10. As shown in Equation (10), the objective function of the CRC-DP method includes two graph constraints which restrict the spatial distribution of samples by Euclidean distance. Here, we verify their effectiveness by executing the CRC-DP method with only fidelity terms and no graph constraints. The comparison results are plotted in Figure 8a, which indicates that the same-class and different-class graph constraints are beneficial for promoting classification accuracy, especially in the case of small sample dimension (m≤6). Limited to the excessive costs of labor and time, it is hard to collect sufficient agricultural data. Therefore, there is usually not enough training data for each kind of disease in practical agriculture production. Here, we conduct an experiment to evaluate the influence of enrollment size (the number of training samples per class) which may seriously affect the performance of the CRC-DP method. In this experiment, τ training samples from each class were randomly selected for training, where τ varies from 100 to 500 with an interval of 50. The testing set is the same as the original testing set described at the beginning of Section 3.2. In total, there are 3τ training samples and 1500 testing samples. The classification accuracies of each method versus different enrollment size are shown in Figure 8b. Note that the identification accuracy is higher than 95% when τ is larger than 175. However, the identification accuracy of the CRC-DP method decreases heavily when the enrollment size is very small (when τ is less than 150, the identification accuracy is smaller than 90%). The reason is that too few training samples per class cannot satisfy CRC’s assumption that the training samples of each class span a subspace and any sample from this class lie on this subspace.

## 4. Conclusions 

A HSI-based early identification method for cucumber leaf diseases is presented and verified through experiments. It builds reconstruction fidelity terms according to the decision rule of CRC and designs a graph constraint based on the label and distribution information. These are fused to steer the offline training procedure of the discriminative projection. Obviously, the method links DR to classification—seeking a low-dimensional space, in which CRC achieves higher identification accuracy and becomes more efficient. Illustrative examples on toy data and wine dataset validate that the offline trained projection is beneficial for the subsequent classification. The experimental results on the hyperspectral data of three cucumber diseases indicate that the CRC-DP method is feasible and effective, which achieves superior identification performance. 

## Figures and Tables

**Table 1:** The identification accuracies and the number of incorrectly diagnosed samples of different methods (for each disease, the quantitative results from top to bottom are the classification accuracy and the number of samples that were wrongly judged, respectively).

Disease	Methods					
	KNN	RF	NB	DA	SVM	CRC-DP
Corynespora Cassiicola	95%	96.4%	92.20	95.00%	95.60%	96.80%
	25	18	39	25	22	16
Cucumber Anthracnose	93.20%	94.6%	95%	94.20%	94.00%	97.80%
	34	27	25	29	30	11
Total	96.07%	97.00%	95.73%	96.40%	96.53%	98.20%
	59	45	64	54	52	27

**Table 2:** Mean online identification time of each query sample for three-class classification.

Methods	KNN	RF	NB	DA	SVM	CRC-DP
Time (*ms*)	0.4454	3.700	0.3463	0.2290	0.0012	0.6537
